# Hemi-nested touchdown PCR combined with primer-template mismatch PCR for rapid isolation and sequencing of low molecular weight glutenin subunit gene family from a hexaploid wheat BAC library

**DOI:** 10.1186/1471-2156-8-18

**Published:** 2007-05-04

**Authors:** Xiu-Qiang Huang, Sylvie Cloutier

**Affiliations:** 1 Cereal Research Centre, Agriculture and Agri-Food Canada, 195 Dafoe Road, Winnipeg, MB R3T 2M9, Canada

## Abstract

**Background:**

Hexaploid wheat (*Triticum aestivum *L.) possesses a large genome that contains 1.6 × 10^10 ^bp of DNA. Isolation of a large number of gene sequences from complex gene families with a high level of gene sequence identity from genomic DNA is therefore difficult and time-consuming. Bacterial artificial chromosome (BAC) libraries can be useful for such work. Here we report on an efficient approach for rapid isolation and sequencing of the low molecular weight glutenin subunit gene family from the 'Glenlea' wheat BAC library via primer-template mismatch PCR using universal primers, primer walking using hemi-nested touchdown (TD) PCR, and followed by direct sequencing of PCR products.

**Results:**

For the primer-template mismatch PCR, the universal primers were designed based on conserved gene coding regions of consensus sequences. The effects of the universal primer-template mismatches on the efficiency of standard PCR amplification were investigated after assembly of sequences from different primers amplifying the same BAC clones. Single or multiple mismatches were observed at 5' terminal, internal and the penultimate position, respectively. These mismatches included the transition mispairs G:T, T:G, A:C and the transversion mispairs A:A, A:G, G:G, G:A. Two or more primer-template mismatches reduced PCR product yield approximately from 2-fold to 10-fold compared to PCR product yield without the primer-template mismatch. For the hemi-nested TD PCR, primers were designed based on the known sequences obtained and/or published. The hemi-nested TD PCR increased both specificity and yield by high and low annealing temperatures in two consecutive amplifications. Comparison of two methods for purifying PCR products prior to sequencing showed that purification using MultiScreen_384_-PCR filter plates had an advantage over ethanol purification because greater numbers of sequencing reactions could be performed from comparable volumes of PCR reactions.

**Conclusion:**

This approach was fast, easy and cost-effective for isolation and sequencing of genes from complex gene families. It may be suitable for (i) isolation of other complex gene families and/or gene homologues from BAC libraries, (ii) for characterization of multi-copy repetitive elements pending availability of BAC libraries, and (iii) for filling in gaps in shotgun BAC sequencing.

## Background

Bacterial artificial chromosome (BAC) libraries are useful for genome analysis, comparative genomics, physical mapping and map-based cloning [1-4]. They can also be used to isolate genes from complex gene families. If a BAC clone contains a single gene, complete gene sequence may be isolated through gene-specific PCR amplification and subsequent primer walking [5] using BAC DNA as template and specific primers close to the end the known sequence obtained. If a BAC clone contains two or more genes that display a high level of gene sequence identity such as the low molecular weight glutenin subunit (LMW-GS) genes, the primer walking using BAC DNA as template is likely not going to succeed due to multiple binding sites of the sequencing primer. PCR amplification is a favorite strategy for isolation of unknown genomic sequences adjacent to known sequences. Several PCR methods are available for this purpose such as inverse PCR [6], ligation-mediated PCR [7], T-linker PCR [8] and TAIL PCR [9]. These methods either require digestion and ligation procedures or are cumbersome, laborious and technically demanding. In addition, they can not ensure specific amplification of individual genes from BAC clones containing two or more genes. Nested PCR [10] is useful in achieving the specificity by the amplification of an extended nucleotide sequence followed by the amplification of a region located within the first amplicon. Touchdown (TD) PCR [11] is a versatile one-step procedure for optimizing PCRs to obtain the specificity even if the degree of primer-template complementarity is not fully known [12]. Here we describe a novel PCR method that combined hemi-nested PCR (a single internal primer) with touchdown PCR for primer-template mismatches. This method is very useful for rapid isolation and sequencing of gene families from BAC libraries.

Wheat gluten is composed of two major groups of proteins, monomeric gliadins and polymeric glutenins. Wheat glutenins consist of high molecular weight glutenin subunits (HMW-GSs) and LMW-GSs. Together, they are the major determinants of wheat bread-making quality. HMW glutenins have been extensively studied because of their importance in dough rheology and their relative ease of analysis at the protein and DNA levels. HMW glutenins represent a small family with a maximum of five of the six subunits expressed, while LMW glutenin genes are part of a much more complex gene family [13].

LMW glutenins represent approximately 40% of the total wheat gluten fraction. LMW-GSs have molecular weight values ranging from 30 KDa to 50 KDa and are about 5–6 times more abundant than HMW-GSs [13] and are therefore the main components of the glutenin polymers. LMW-GSs are encoded at the *Glu-A3*, *Glu-B3 *and *Glu-D3 *loci on the short arms of chromosomes 1A, 1B and 1D, respectively. The number of LMW glutenin-like genes in hexaploid wheat was estimated to range from 30 to 40 based on Southern hybridization [14]. These LMW-GS genes were classified into three types, LMW-i, LMW-m and LMW-s, based on the first amino acid residue of their N-terminal sequences, which correspond to isoleucine, methionine and serine, respectively [13]. However, LMW glutenins have been less studied because of their abundance and the inherent difficulty to characterize them at the protein level i.e. SDS-PAGE. The loci are complex with many subunits encoded at each locus and the presence of pseudogenes.

'Glenlea' is a Canadian Western Extra Strong (CWES) spring wheat variety. The LMW-i and LMW-s type genes in 'Glenlea' have been recently cloned using genomic DNA and developing seed cDNA libraries [15,16]. To date, more than 90 cDNA and genomic DNA clones of LMW-GS genes have been published [17]. Most studies reported only a single or very few genomic DNA clones of LMW-GS genes that were isolated from a given wheat variety due to their complex compositions [15,16]. To further our understanding of the LMW-GS gene family, a BAC library was constructed from 'Glenlea' [18]. Here we report an efficient method for rapid isolation of sequences of the complex LMW-GS gene family from the 'Glenlea' BAC library using universal primers for primer-template mismatch PCR, primer walking using hemi-nested touchdown PCR and direct sequencing of PCR products.

## Results

### Identification of BAC clones containing LMW-GS genes

A total of 383 positive BAC clones were identified by screening the 24 high-density filters with the LMW-GS composite probe. Positive signals may include BAC clones containing gliadin sequences, because LMW-GS and gliadin sequences share approximately 60% similarities [13].

**Figure 1:**
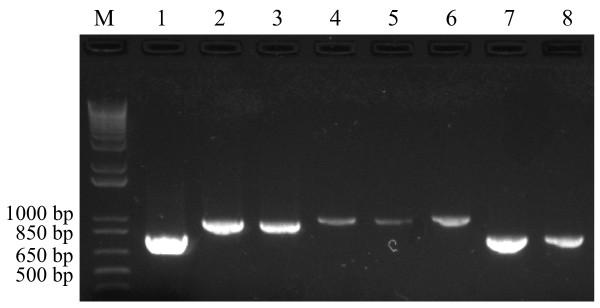
**Amplification of eight representative BAC clones with primer pair LMWF3/LMWR4 shows variable fragment sizes**. Amplicon intensity represents various primer-template mismatches. M, 1 kb plus DNA ladder; lanes 1–8, PCR amplification of TaE0503E07, TaE1238L16, TaE0154F22, TaE0359D24, TaE01157C20, TaE0709A09, TaE0099E23 and TaE0072P19, respectively.

In order to confirm the identity of positive BAC clones containing LMW-GS genes, LMW-GS specific primers were designed. After PCR screening, 72 out of the 383 BAC clones produced one amplicon with at least one of the three primer pairs. Thirty-one BAC clones were identified by single primer pair, while 41 BAC clones were identified by using two or three primer pairs (Table 1). Fig. 1 shows eight representative BAC clones identified using the primer pair LMWF3/LMWR4.

**Table 1:** Number of BAC clones producing single amplicons using three primer pairs alone or in combination.

Primer pair(s)	Number of BAC clones producing single amplicons
LMWF3/LMWR4 (PP1)	16
LMWF10/LMWR9 (PP2)	8
LMWF11/LMWR11 (PP3)	7
PP1 and PP2	4
PP1 and PP3	22
PP2 and PP3	0
PP1, PP2 and PP3	15
Total	72

### Clean-up and direct sequencing of PCR products

In order to remove the remaining salts, unincorporated dNTPs and primers from the PCR reactions, we compared two PCR purification methods, i.e. MultiScreen_384_-PCR filter plate and ethanol precipitation, for their ability to produce large amounts of high quality template for sequencing reactions. The PCR product from a single PCR reaction purified using the ethanol precipitation was only sufficient for a single sequencing reaction, whereas the combined PCR product from two PCR reactions purified using the MultiScreen_384_-PCR filter plate was sufficient for five (PCR with primer-template mismatches, see below) to 16 (PCR without primer-template mismatch) sequencing reactions. Hence, the Multiscreen_384_-PCR filter plate provided better recovery of PCR products and was particularly efficient for weak amplifications with primer-template mismatches. Both PCR purification methods produced 100% successful sequencing reactions. The length of high quality sequences obtained using ethanol precipitation and MultiScreen_384_-PCR filter plate was 530 and 548 bases, respectively.

Given the large number of PCR products to be sequenced, we employed a direct sequencing approach using the same primers used for the PCR reactions. Direct sequencing of PCR amplicons is efficient and cost-effective. Therefore, direct sequencing is advantageous over the cloning approach which is time-consuming and costly.

The PCR products of the 72 BAC clones amplified using the three primer sets were directly sequenced. Single sequence length with Phred score greater than 20 could reach up to 630 bases with an average of 535 bases. A single LMW-GS sequence was isolated in 23 BAC clones using one of the three individual primers, whereas 29 BAC clones had two or three single sequences isolated by different primers. This indicated that the 29 BAC clones might contain a single or two LMW-GS gene(s), when different sequences were assembled into one or two contigs. The remaining 20 BAC clones had a LMW-GS pseudogene sequence or two LMW-GS sequences isolated by a single primer pair, which resulted in overlapping sequences. For these BAC clones with the overlapping sequences using one pair of primers, specific primers need to be designed to amplify individual LMW-GS genes.

### Nucleotide sequence alignment

**Figure 2:**
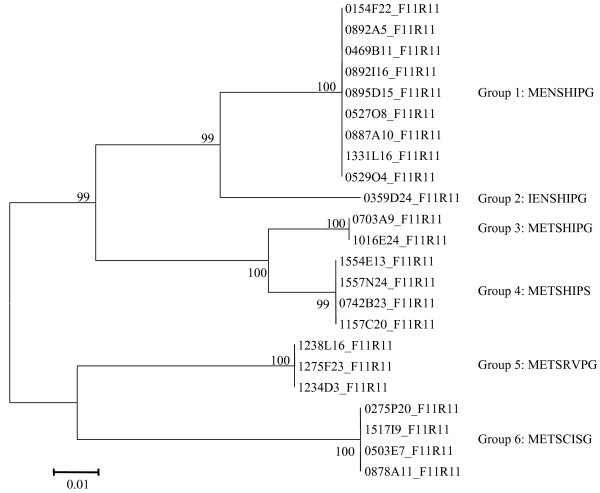
**Phylogenetic tree of partial LMW-GS gene sequences using primer pair LMWF11/LMWR11 constructed using MEGA3.1**. Bootstrap values are indicated; bootstrap percentages are based on 1000 iterations. The scale bar indicates the level of sequence divergence. DNA sequences with the same N-terminal amino acid sequence were clustered in the same group.

Twenty-three high quality sequences of partial LMW-GS genes were isolated using the primer set LMWF11/LMWR11. Their length ranges from 556 to 711 bases with an average of 628 bases. A phylogenetic tree of the 23 sequences was constructed using MEGA3.1. The 23 sequences were clustered into six groups, which correspond to six different N-terminal sequences of LMW-m and LMW-s type genes (Fig. 2). Sequence alignment showed that the sequences from different BAC clones in the same groups were identical in the aligned regions, suggesting that these BAC clones may contain identical LMW-GS genes.

### Hemi-nested TD PCR for primer walking

**Figure 3:**
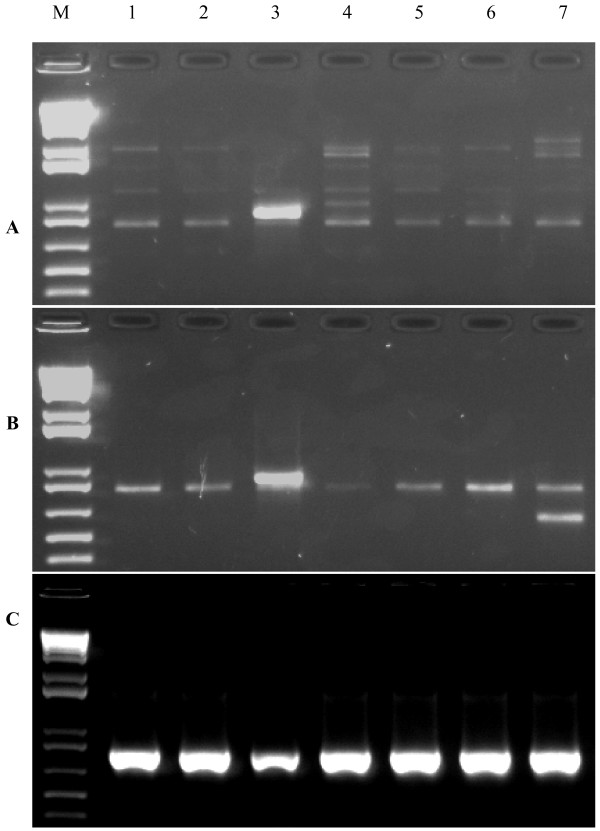
**Comparison of standard PCR with hemi-nested touchdown (TD) PCR**. (A) Standard PCR with primers LMWF38d/R19b and annealing temperature of 50°C, (B) the first round TD PCR using primers LMWF38d/R19b and (C) the second round hemi-nested TD PCR using primers LMWF38d/R19c. In the TD program, the annealing temperature is progressively lowered from 65°C to 50°C by 0.5°C every cycle, followed by 15 additional cycles at 50°C. M, 1 kb plus DNA ladder; lanes 1–7, PCR amplification of TaE0879B17, TaE0877F13, TaE0742B23, TaE0469B11, TaE0428D3, TaE0356P04 and TaE0212J01, respectively.

After we obtained partial sequences of the coding regions of LMW-GS genes from individual BAC clones using the universal primers, we first tried to perform primer walking using BAC DNA as template and specific primers close to the end the known sequence obtained. This method worked well for BAC clones containing single LMW-GS gene in many cases, but not for BAC clones containing two or more LMW-GS genes (data not shown). Then we tried to use TAIL PCR for the BAC clones containing two or more LMW-GS genes by designing three specific primers based on the known sequences obtained and one degenerated primer as described in Liu and Huang [9]. In many cases, either more than two PCR products were amplified or no amplicons were generated (data not shown). Thirdly, we designed primers based on 5' and 3' terminal sequences of the known LMW-GS gene sequence [GenBank: X13306] [19] combined with the above mentioned specific primers in order to generate PCR fragments from these BAC clones by primer-template mismatches. This method did not work for all BAC clones even though we optimized PCR conditions by decreasing the concentration of Mg^+2 ^or/and using different annealing temperatures. After numerous attempts, we finally developed a novel PCR method, hemi-nested TD PCR combined with primer-template mismatches, for primer walking. As shown in Fig. 3A, standard PCR of seven BAC clones using the forward primer LMWF38d and the external reverse primer LMWR19b with annealing temperature at 50°C generated multiple unspecific amplicons. Standard PCR using the same primers with annealing temperature at 60°C did not produce amplification at all (data not shown). Fig. 3B shows the first round TD PCR amplification using a program in which the annealing temperature is progressively lowered from 65°C to 50°C by 0.5°C every cycle, followed by 15 additional cycles at 50°C. Faint single PCR product was observed for five BAC clones and two PCR amplicons were generated for BAC clone TaE0212J01. After the second round hemi-nested TD PCR using the same forward primer LMWF38d and the internal reverse primer LMWR19c with the same TD program, a single strong PCR product was amplified for the all seven BAC clones (Fig. 3C). When the amplicons of Figure 3A were amplified using the same primers and PCR conditions, the identical PCR products were observed but they were weaker than the amplicons in Figure 3C (data not shown). The hemi-nested TD PCR worked well for all the BAC clones irrespective of the presence of one or two LMW-GS genes. So far, two different LMW-GS gene sequences have been obtained from four BAC clones, respectively.

## Discussion

We regularly use BAC pool screening to identify BAC clones from our library. This method is quite efficient for single or low copy target. Our library contains more than 650000 clones and can be screened by this method using only 221 PCR reactions. Filter hybridization is however much more efficient for large gene family such as the LMW-GS gene family described in this study. Considering that nearly 400 BAC clones were identified, would translate in the identification of more than 300 Super Plate Pools (SPP). The screening of the Plate Pools, Row Pools and Column Pools for such a large number of SPP would be far more labour intensive than filter hybridization in this case. Since LMW-GS genes have about 60% sequence similarities with gliadin genes [13], we identified positive BAC clones containing LMW-GS genes using hybridization screening followed by PCR validation. Of the 383 BAC clones identified by the filter hybridization, 72 were confirmed to have LMW-GS gene(s) using three LMW-GS gene-specific primers (Table 1).

### Direct sequencing of PCR products amplified by mismatch primers

PCR amplifications of most BAC clones were generated by mismatch primers (see below). Direct sequencing of these PCR amplicons is also advantageous over the cloning approach, because the mismatch primers do not exist in the sequences obtained using direct sequencing, but in the sequences obtained using M13 forward and reverse primers for plasmid DNA from the cloning method. In this case, the sequences from the cloned PCR products using the mismatch primers will affect the sequence assembly from different PCR products unless the mismatch primers are removed before sequence assembly. If primers amplify multiple fragments with different sequences from one BAC clone, direct sequencing is not useful. Cloning the multiple fragments into separate colonies makes it possible to sequence them separately.

### Primer-template mismatches

PCR amplification requires two primers complementary to the beginning and the end of the DNA fragment of the template in opposite orientation. When genomic DNA is used for PCR amplification, the primers first bind the template with complementarity (perfect match). If complementary template is not present, the primers may bind the template with non-complementarity (mismatch). By utilizing this strategy, homologous sequences from different varieties or homoeologous sequences from different related species may be successfully amplified and cloned. BAC DNAs with their 100–150 kb sequences have reduced complexity when compared to genomic DNAs and a perfect match to given primers may not be present. LMW-GS genes share approximately 80% identity [13]. As a consequence, universal primer pairs can be designed based on conserved regions of consensus sequences and used to amplify a number of LMW-GS genes using different BAC DNA as templates.

The present study confirmed this hypothesis. Using BAC DNA as template and primer-template mismatches, the primer set LMWF11/LMWR11 designed from LMW-s type gene (AY542898) [20] could amplify LMW-m type genes (Fig. 2). Similarly, the primer set LMWF3/LMWR4 designed from LMW-m type genes [20] could amplify LMW-s type genes (see below). The primer set LMWF10/LMWR9 designed from LMW-i type genes [15,20] could amplify all three gene types, LMW-i, LMW-m and LMW-s (data not shown).

Several studies have investigated the effects of primer-template mismatches at the 3' terminus of one primer on the efficiency of PCR amplification [21-25]. Assembly of sequences from different primers amplifying the same BAC clones allows us to make detailed analysis of the primer-template mismatches between the primers LMWF3/LMWR4 and eight different BAC clones (Table 2). Eight different LMW-GS genes, corresponding to LMW-m and LMW-s types, were isolated using the primer set LMWF3/LMWR4. Single or multiple mismatches were observed at 5' terminal, internal and the penultimate position, respectively. These mismatches included the transition mispairs G:T, T:G, A:C and the transversion mispairs A:A, A:G, G:G, G:A. The investigation of Kwok et al. [22] indicated that single internal mismatches had no significant effect on PCR product yield, while those at the 3'-terminal base significantly reduced PCR product yield. In the present study, the reduction of PCR product yield correlated with the number of the primer-template mismatches. The primer LMWF3 has two internal mismatches with the BAC clone TaE0099E23 as template, whereas the primers LMWF3 and LMWR4 have one penultimate mismatch from the 3'-terminus and one internal mismatch, respectively, with the template of the BAC clone TaE1238L16 (Table 2). Two primer-template mismatches reduced PCR product yield approximately 2-fold, three or more primer-template mismatches reduced PCR product yield about up to 10-fold, as compared to PCR product yield of TaE0503E07 without the primer-template mismatch (Table 2 and Fig. 1). A different signal intensity occurred in the BAC clones TaE1157C20 (sample 5) and TaE0703A09 (sample 6) in Figure 1, even though these two clones have the same mismatsches (Table 2). A possible explanation is the insertion size of TaE1157C20 being larger than that of TaE0703A09, which resulted in the amount of initial target template of TaE1157C20 being relatively smaller than that of TaE0703A09 with the same amount (ng) of BAC DNA. A primer-template mismatch at the 3'-terminus was not found in this study. The effect of the primer-template mismatch at the 3'-terminus of one or both primers on PCR amplified for LMW-GS genes could be investigated by deliberately introducing base alteration at the 3'-terminus of synthesized primers.

**Table 2:** Position (base in bold) and type of paired base mismatch(es) between primer pair LMWF3/LMWR4 and eight BAC clone templates.

BAC clone (template)	LMWF3 (5'→3') GACAAGTGCCATTGCACAGA	Primer-template mismatch	LMWR4 (5'→3') GCTGTACAACGGCACATTGA	Primer-template mismatch	N-terminal sequence
TaE0503E07	GACAAGTGCCATTGCACAGA	No mismatch	GCTGTACAACGGCACATTGA	No mismatch	METSCISG
TaE1238L16	GACAAGTGCCATTGCACA**A**A	G:T^a^	GCTGTACAACGGCAC**G**TTGA	A:C	METSRVPG
TaE0154F22	GACAAGTGCCATTGCACA**A**A	G:T	**T**CT**A**TACAACGGCACATTGA	G:A, G:T	MENSHIPG
TaE0359D24	**A**ACAAGTGCCATTGCACA**A**A	G:T (2×)	**T**CTGTACAACGGCACATTGA	G:A	IENSHIPG
TaE1157C20	GACAAGTGCCATTGCACA**A**A	G:T	GC**C**GTACA**C**CGGCAC**G**TTGA	T:G, A:G, A:C	METSHIPS
TaE0703A09	GACAAGTGCCATTGCACA**A**A	G:T	GC**C**GTACA**C**CGGCAC**G**TTGA	T:G, A:G, A:C	METSHIPG
TaE0099E23	GACA**TC**TGCCATTGCACAGA	A:A, G:G	GCTGTACAACGGCACATTGA	No mismatch	METSCIPG
TaE0072P19	GACA**TC**TGCCATTGCACAGA	A:A, G:G	GCTGTACAACGGCACATT**A**A	G:T	METSCIPG

^a^The first base from the primer and the second base from complementary sequence of template.

### Hemi-nested TD PCR for primer walking

Both nested PCR and TD PCR can produce specific fragments [10,11]. Nested PCR was widely used to detected specific viral DNA sequences in clinical specimens [26]. TD PCR has been used to eliminate multiple unwanted products in which the degree of primer-template complementary is not fully known [12]. High and low annealing temperatures in TD PCR could increase both specificity and yield [12]. In the present study, we developed a novel PCR technique for primer walking, hemi-nested TD PCR, which combines nested PCR with TD PCR for primer-template amplification. Compared with other PCR method for primer walking, the hemi-nested TD PCR has many advantages: (i) a simple protocol without any digestion and ligation steps; (ii) high specificity; and (iii) high yield of PCR products, which can be directly sequenced.

Design of universal primers based on primer-template mismatches for the identification of all positive BAC clones containing LMW-GS genes is a useful approach towards isolation of all LMW-GS gene sequences from a wheat BAC library. Complete LMW-GS gene sequences will be obtained by primer walking using the hemi-nested touchdown PCR and direct sequencing of amplified PCR products using the same primers. So far, 12 complete LMW-GS gene sequences including their untranslated 5' and 3' flanking regions from different BAC clones have been obtained using the above described procedures (data not shown). This work is in progress and all LMW-GS gene sequences will be isolated from the 'Glenlea' BAC library in the near future.

## Conclusion

In summary, this paper describes a detailed and efficient method for rapid isolation of LMW-GS gene sequences from the wheat 'Glenlea' BAC library using filter hybridization followed by primer-template mismatch PCR combined with hemi-nested touchdown PCR and direct sequencing of PCR products. This approach was fast, easy and cost-effective. It may be suitable for (i) isolation of other complex gene families and/or gene homologues from BAC libraries, (ii) for characterization of multi-copy repetitive elements pending availability of BAC libraries, and (iii) for filling in gaps in shotgun BAC sequencing.

## Methods

### BAC library screening

The Glenlea BAC library contains 656 640 clones with 3.1× haploid genome coverage and has been gridded onto 24 high-density filters [18]. A composite probe, consisting of the complete coding regions of a LMW-i [GenBank:AY542896] [15], a LMW-m [GenBank: AY542897] and a LMW-s [GenBank:AY542898] [20], was labeled with [α-^32^P]dCTP using Ready-to-Go DNA labeling beads (Amersham Biosciences, Piscataway, NJ, USA) and used to screen the 24 high-density filters. Hybridization was performed as described in Nilmalgoda et al [18].

### BAC DNA extraction

Positive BAC clones identified by high-density filter hybridization were inoculated in 96-well plates containing 1.5 mL of 2× YT supplemented with 12.5 μg chloramphenicol/ml and were grown in a C25KC incubator shaker (New Brunswick Scientific Co., Edison, NJ, USA) at 37°C and 300 rpm for 20 h. BAC DNA was isolated and purified using an Eppendorf Perfectprep BAC 96 purification kit (Hamburg, Germany) adapted for a liquid handling robot (Qiagen 3000, Mississauga, ON, Canada). BAC DNA was eluted in a final volume of 60 μl.

### LMW-GS gene-specific primer design and PCR

LMW-GS gene-specific primers were designed using Primer3 [27]. The primer pairs LMWF3/LMWR4 and LMWF10/LMWR9 were designed based on the coding regions of consensus sequences of full-length LMW-m and -i type cDNA clones, whereas the forward primer LMWF11 and the reverse primer LMWR11 were from the promoter region and the repetitive domain of genomic sequence of LMW-s type AY542898 (Table 3) [16]. These primers were used to amplify LMW-GS genes using the BAC clones identified by hybridization and 'Glenlea' genomic DNA as templates.

**Table 3:** LMW-GS gene specific primers designed based on consensus sequences of full-length LMW-GS cDNA clones and a genomic clone.

Primer	Primer sequence	Contig no.^a^	Tm (°C)	Product size (bp)
LMWF3	GACAAGTGCCATTGCACAGA	5, 3	60	
LMWR4	GCTGTACAACGGCACATTGA	5, 3	60	820
LMWF10	TCACAGCAACAACAACCACA	7	60	
LMWR9	CTATCTGGTGTGGCTGCAAA	7	60	784
LMWF11	CCAAACTCGGTTGCAAAAGT	AY542898	60	
LMWR11	TGGTGGTTGTTGCGGTAGTA	AY542898	60	805

^a^Contig number from Özdemir and Cloutier [20].

The PCR reactions used either 5–10 ng BAC DNA or 200 ng genomic DNA as template, 1× PCR buffer, 1 mM MgCl_2 _(to increase the stringency of the reactions), 0.8 mM dNTPs, 0.6 μM of each primer, 0.1 μl of 10× BSA (1 mg/ml) and 1 Unit *Taq *DNA polymerase made up to a final volume of 25 μl with sterile ddH_2_O. The PCR reaction was carried out in a PTC-100 thermocycler (MJ Research Inc., Waltham, MA, USA). Following 5 min of denaturation at 94°C, 35 cycles were performed with 30 s at 94°C, 30 s at 60°C, 90 s at 72°C, and a final extension step of 10 min at 72°C. PCR products were separated on a 1.5% agarose gel in 0.5× TBE buffer. The gel was photographed and PCR band intensity was measured using the spot densitometry analysis tool of an AlphaImager HP imaging system (Alpha Innotech Corp., San Leandro, CA, USA). The amplicons were quantified by comparing their pixel intensity values designated density value to reference samples which in this case were lambda *Hind*III fragments of known concentrations.

### Primer walking by hemi-nested touchdown (TD) PCR

For primer walking, the forward primer LMWF38d (5'-TATCACTCCACCTCAGCATTG-3') was designed from the promoter region of a previously cloned LMW glutenin gene [LMWG-1D1; GenBank: X13306] [19]. The external (LMWR19b: 5'-TGTTGTTGTGGAGGTAAAACTA-3') and internal (LMWR19c: 5'-GAAAATGGTGGTTGTTGCTG-3') reverse primers were designed based on the sequences obtained from the PCR product of BAC clone TaE0879B18 amplified using the primer pair LMWF3/LMWR4. The standard PCR reaction was the same as the above, except that 1.5 mM instead of 1 mM MgCl_2 _and annealing temperature at 50°C were used. The forward primer LMWF38d and the external reverse primer LMWR19b were used for the first round TD PCR. The PCR reaction was the same as the above mentioned standard PCR. Following 5 min of denaturation at 94°C, samples were subjected to 30 cycles in a TD program (94°C for 30 s, 65°C for 30 s and 72°C for 90 s, followed by a 0.5°C decrease of the annealing temperature every cycle). After completion of the TD program, 15 cycles were subsequently performed (94°C for 30 s, 50°C for 30 s and 72°C s for 90 s) ending with a 10 min extension at 72°C. One microliter of the first round TD PCR product was diluted with 199 μl distilled water and 1 μl of the diluted DNA was added to the second round hemi-nested TD PCR mixture. The same forward primer LMWF38d and the internal reverse primer LMWR19c as well as the same TD program were used for the hemi-nested TD PCR amplification.

### Purification of PCR products

#### Purification of PCR products using MultiScreen_384_-PCR filter plates

Purification of PCR products was performed using MultiScreen_384_-PCR filter plates following manufacturer's introduction with some modifications (Millipore Corp., Billerica, MA, USA). A total of 50 μl from two 25 μl PCR reactions were transferred to a single well of a MultiScreen_384_-PCR filter plate. The MultiScreen_384_-PCR filter plate was placed on a MultiScreen vacuum manifold (Millipore Corp., Billerica, MA, USA) and the vacuum was applied for 20 min at 10 inches of Hg, or until the wells were completely empty. The plate was removed from the vacuum manifold and any remaining droplets were blotted from the bottom of the filter with a paper towel. Milli-Q-grade water (65 μl) was added to each well and the above steps were repeated. Then Milli-Q-grade water (52 μl) was added to each well and the samples were resuspended on a plate shaker at low speed for 10 min. After mixing by pipetting up and down, the purified product was transferred from MultiScreen_384_-PCR filter plate to 96-well PCR plate for storage. The concentration of the purified product was estimated on 1.5% agarose gel in 0.5× TBE buffer using a 2 μl aliquot of sample added to 10 μl of water and 2 μl of 6× loading buffer.

#### Purification of PCR products by ethanol precipitation

Alternatively, PCR products can be purified using ethanol precipitation. Each well (25 μl reaction) of the 96-well PCR plate had 4.2 μl of 3 M sodium acetate (pH 5.2) and 60 μl of pre-chilled 95% ethanol (-20°C) added. The plate was placed in a freezer at -80°C for 12 min and then centrifuged at 1900 × *g *for 45 min. After supernatant was discarded by gently inverting the plate, the plate was centrifuged upside down on a paper towel at 40 × *g *for 1 min to remove the remaining ethanol. The pellets were washed with 120 μl of 70% ethanol. The plate was centrifuged at 1900 × *g *for 12 min and the ethanol was discarded by gently inverting the plate. The plate was centrifuged upside down on a paper towel at 40 × *g *for 1 min and then dried in the laminar flow hood for 10 min. The dried, purified PCR product is ready to be used as template in sequencing reactions.

### Sequencing of PCR products

Sequencing reactions contained 40–50 ng PCR product as template, 1 μl for ABI 384-well plate (1.5 μl for 96-well PCR plate in parenthesis, thereafter the same) of 5× sequencing buffer, 1 μl (1 μl) of 5.2 μM primer (primers as used in PCR reactions), and 0.4 μl (1 μl) of BigDye reaction mix in a total volume of 6 μl (10 μl). The sequencing reaction was carried out using an ABI 384-well plate in a PTC-200 DNA engine thermocycler or a 96-well PCR plate in a PTC-100 thermocycler (MJ Research, Inc., Waltham, MA, USA). Following 5 min of denaturation at 94°C, 60 cycles were performed with 10 s at 94°C, 10 s at 55°C, 4 min at 60°C, and a final extension step of 10 min at 60°C.

### Purification of sequencing reactions using ethanol precipitation

We added 4.8 μl (8 μl) of double distilled water and 19.2 μl (32 μl) of 95% ethanol to each well of the 384-well plate (96-well plate) and then mixed thoroughly by pipetting up and down. The plate was incubated in the dark at room temperature for 30 min and then centrifuged at 1900 × *g *for 45 min. After discarding supernatant, the plate was centrifuged upside down on a paper towel at 40 × *g *for 1 min. The pellets were washed with 35 μl (60 μl) of 70% ethanol and the plate was centrifuged at 1900 × *g *for 15 min. After removing the washing solution, the plate was centrifuged upside down on a paper towel at 40 × *g *for 1 min and then dried in the laminar flow hood (in dark) for 15 min. Ten microliters of Hi-Di formamide (Applied Biosystems, Foster City, CA, USA) was added to each well and then the plate was centrifuged briefly, denatured in a PTC-200 or PTC-100 thermocycler at 95°C for 5 min and chilled on ice. The PCR products were sequenced on a 3100 Genetic Analyser (Applied Biosystems).

### Nucleotide sequence analysis

DNA sequences were processed and assembled using our in-house developed software called SOOMOS v0.6 (Banks, personal communication). Base calling, quality score generation and low quality sequence removal were performed with the software PHRED [28] using the '-trim_alt' option. The software CAP3 [29] was used to assemble the processed reads into contiguous sequences (contigs). DNA sequence multiple alignment, translation and identification of open reading frames (ORFs) were conducted using CLUSTAL W v1.82 [30] and DNAMAN v3.2 (Lynnon Corp., Vaudreuil-Dorion, Quebec, Canada). MEGA3.1 [31] was used to construct a phylogenetic tree using the Neighbor-joining method. Bootstrap tests were performed using 1000 replications.

## Abbreviations

BAC = bacterial artificial chromosome; BSA = bovine serum albumin; CS = Chinese Spring; dNTP = deoxyribonucleotide triphosphate; HMW-GS = high molecular weight glutenin subunit; LMW-GS = low molecular weight glutenin subunit; PCR = polymerase chain reaction; TBE = tris borate EDTA; TD = touchdown; YT = yeast tryptone.

## Authors' contributions

XQH designed and carried out the experiments, performed the sequence alignment and the data analysis, and drafted the manuscript. SC conceived the study, participated in the data analysis, and revised the manuscript. Both authors read and approved the final manuscript.
